# Reply: Value of tyrosinase RT–PCR in melanoma prognosis

**DOI:** 10.1038/sj.bjc.6600835

**Published:** 2003-03-18

**Authors:** H Gogas

**Affiliations:** 1 Lecturer in Medical Oncology, 1st Department of Medicine, PO Box 14120 Athens, 11510, Greece

**Sir**,

In the comment of Dr Konstantopoulos ‘Gogas *et al*, give no information pertaining to the status of melanoma cells circulating in their patients before any surgical manipulation (i.e. at first presentation). In our work (Gogas *et al*, 2002), in 27 patients tested, one was already positive before operation becoming negative some time following operation’ we would like to state that more than 95% of the patients referred to our Center have already been diagnosed with melanoma and require wide local excision, sentinel lymph node and consideration for adjuvant treatment. So, it was not possible to obtain blood from patients before any surgical manipulation. Perhaps, they should define if this was the case in the 27 patients they studied in the work they have published.

Concerning the comment ‘This type of information may permit further confirmation of a theory claiming that surgery manipulations may cause forced release of melanocytes to blood circulation’ in our discussion, we argue that even though this is not a randomised study and there were no untreated patients, and one could speculate that the conversion was a consequence of greater time after surgery, the surgery in this case either dislodging tumour cells or removing the source of tumour cells, the lower probability of recurrence strongly argues that the reversion was a consequence of interferon therapy.

Finally, I cannot reconcile why Dr Konstantopoulos believes that ‘for a more reliable conclusion to be drawn, as interferon administration could possibly induce transcription of tyrosinase gene, one should also include subjects under similar doses of interferon for other purposes as controls’. The regiment used in our study is only approved by the FDA for high-risk melanoma patients.
